# Pulmonary light chain deposition disease secondary to Sjögren disease: a case report

**DOI:** 10.3389/fimmu.2026.1747455

**Published:** 2026-02-16

**Authors:** Xiaotong Xu, Lingjian Wang, Chunsheng Zhou, Lu Zhang, Ting Zhang, Min Peng, Rui’e Feng, Juhong Shi

**Affiliations:** 1 Department of Respiratory and Critical Care Medicine, Peking Union Medical College Hospital, Chinese Academy of Medical Sciences & Peking Union Medical College, Beijing, China; 2 4 + 4 Medical Doctor Program, Chinese Academy of Medical Sciences & Peking Union Medical College, Beijing, China; 3 Department of Hematology, Peking Union Medical College Hospital, Chinese Academy of Medical Sciences & Peking Union Medical College, Beijing, China; 4 Department of Pathology, Peking Union Medical College Hospital, Chinese Academy of Medical Sciences & Peking Union Medical College, Beijing, China

**Keywords:** bortezomib, case report, hemoptysis, light chain deposition disease, pulmonary cysts, pulmonary nodules, Sjögren disease

## Abstract

Pulmonary light chain deposition disease (PLCDD) is a rare disorder characterized by the deposition of immunoglobulin light chains in the lungs, often associated with lymphoplasmacytic proliferative and autoimmune disease such as primary Sjögren disease (pSjD). Due to its rarity, there is currently no established definitive management of PLCDD. In this case report, we present the clinical presentation of a 42-year-old female with a history of pSjD who experienced recurrent hemoptysis over a period of 3 years. Radiological and pathological assessments confirmed pulmonary involvement of light chain deposition disease. Despite initial failure of glucocorticoids and immunosuppressive agents in targeting pSjD, subsequent combination therapy with bortezomib and dexamethasone (BD) resulting in significant clinical and radiological improvement. The successful use of this combination treatment in PLCDD represents a significant breakthrough, highlighting the potential effectiveness of targeted therapies for PLCDD secondary to autoimmune disease.

## 1 Introduction

Light chain deposition disease (LCDD) is a rare disorder characterized by the accumulation of non-amyloid immunoglobulin light chains in various organs or tissues, resulting in structural and functional damage. Pulmonary involvement is rare and frequently complicated by lymphoplasmacytic or autoimmune disorders (1). The clinical presentation of PLCDD varies widely, ranging from asymptomatic presentations to respiratory failure (2, 3). Due to its broad clinical manifestation and limited number of cases, the optimal treatment for PLCDD remains to be definitively established. We report a case of PLCDD patient who presented with recurrent hemoptysis and experienced significant improvement in her condition following a combined therapy regimen of bortezomib and dexamethasone.

## 2 Case presentation

The patient was a 42-year-old woman presented with a three-year history of progressively worsening hemoptysis, which evolved from blood-streaked sputum to the daily expectoration of several tens of milliliters of fresh blood, accompanied by a productive cough. She was a non-smoker with a history of pSjD.

**Figure 1:**
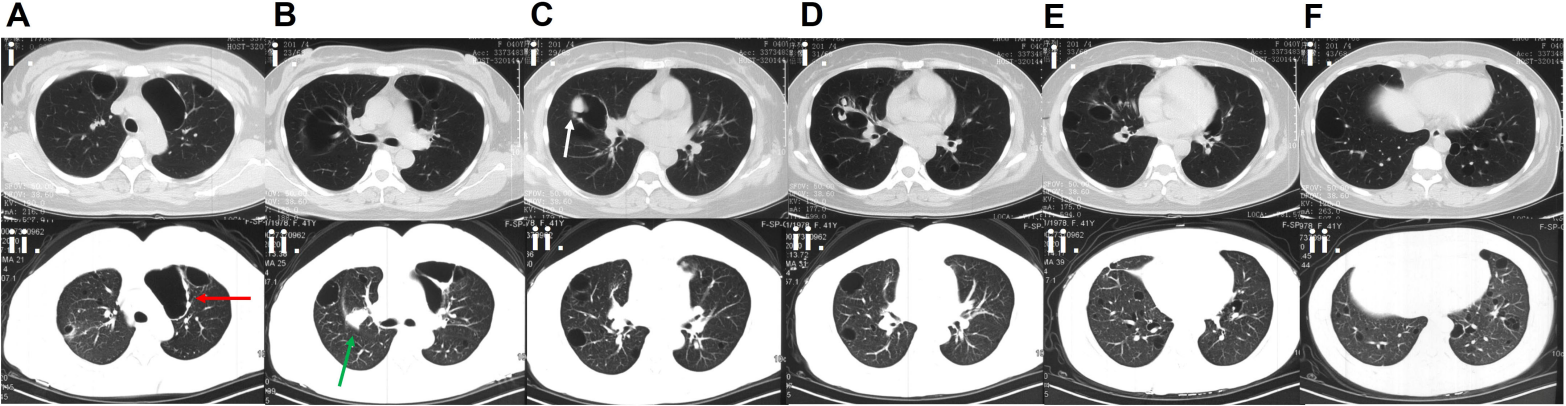
CT scans at presentation and during follow-up. **(Ai–Fi)** Chest CT scan without contrast in November 2018, showing multiple cysts in both lungs, with a solid nodule (indicated by the white arrow) observed within the cyst in the middle lobe of the right lung. **(Aii–Fii)** Chest CT scan with contrast in April 2020 showing multiple air-containing cavities in both lungs, with wall thickening and small nodules (indicated by the red arrow) developing in the left upper lobe, and a nodule (indicated by the green arrow) adjacent to the mediastinum in the right lung.

Since 2009, the patient had persistent daily symptoms of xerophthalmia and xerostomia, accompanied by recurrent salivary gland swelling and the need to drink fluids to swallow dry food. In March 2018, the development of recurrent hemoptysis led her to seek systematic medical evaluation at a local hospital. The physicians at the local hospital noted the patient’s longstanding symptoms of xerophthalmia and xerostomia. Serological testing was performed and revealed positive anti-SSA antibodies. Salivary gland scintigraphy demonstrated poor visualization of both parotid and submandibular glands, indicating severe functional impairment. In November 2018, the initial chest computerized tomography (CT) scan at a local hospital revealed multiple cysts in both lungs, with a solid nodule observed within the cyst in the middle lobe of the right lung (**Figures 1Ai-Fi**). To further establish the diagnosis, in December 2018, video-assisted thoracoscopic surgery (VATS) was performed at a local hospital to obtain lung biopsies from middle lobe and upper lobe of the right lung. However, histological examination of the lung biopsy only indicated granulomatous inflammation. Considering that the patient met four of the six items of the 2002 American-European Consensus Group (AECG) classification criteria (4), she was diagnosed with pSjD-associated pulmonary involvement in June 2019. Hydroxychloroquine (HCQ) 0.2 g daily was administered to control her underlying disease. After 4 months of treatment, she returned to the local hospital complaining of worsened hemoptysis, which had progressed to substantial volumes of fresh blood. The chest CT scan in April 2020 revealed thin-walled cysts diffusely distributed in both lungs (**Figures 1Aii-Fii**). Her treatment plan was modified to include prednisone 30 mg daily for one month and followed by gradual tapering to 7.5 mg daily along with mycophenolate mofetil (MMF) 0.5 g daily and HCQ 0.2 g daily. Her symptom of hemoptysis partially improved after this treatment. The patient visited our hospital for further treatment in March 2021.

On examination, the patient was afebrile with pulse rate of 68/min. Her BP was 110/70 mmHg. Diminished breath sound was heard at right lung, but no rale was noticed on auscultation. She had a normal heart rhythm. No pathological murmur was heard. Her abdomen was nontender. The remaining physical examination was unremarkable.

Laboratory studies revealed normal complete blood count, renal function, liver function. The serum calcium level was 2.28 mmol/L (reference range: 2.13-2.70). Routine urinalysis was normal. However, an elevated erythrocyte sedimentation rate of 23 mm/h (reference range: 0-20) was observed, along with a high-sensitivity C-reactive protein level of 4.64 mg/L (reference range: <3), increased serum IgG level of 19.06 g/L (reference range: 7.00-17.00). Results from serum protein electrophoresis (SPEP), serum immunofixation electrophoresis (SIFE), and urine immunofixation electrophoresis (UIFE) were all negative. Serum free light chain (sFLC) analysis revealed an elevated λ level (32.0 mg/L; reference range: 5.7-26.3) with a normal κ/λ ratio (0.528), suggesting a polyclonal or reactive process rather than monoclonal expansion. Furthermore, PET-CT showed no evidence of osteolytic lesions, and the bone marrow aspirate showed no morphological evidence of clonal plasma cell infiltration. Extensive serological tests revealed positive results in antinuclear antibodies at a titer of 1:160 (reference range: <1:80), anti-SSA antibodies, and anti-Ro52 antibodies. Pathogen analysis of the bronchoalveolar lavage fluid was negative.

Pulmonary function tests indicated a mildly obstructive ventilatory disorder, evidenced by an FEV1/FVC ratio of 68.8%. Total lung capacity (TLC) was within the normal range at 94% of the predicted value; while diffusing capacity for carbon monoxide (DLCO) was moderately reduced to 64% of predicted. Additionally, there was evidence of small airway dysfunction, with maximum expiratory flow rates (MEF_75_, MEF_50_, MEF_25_) markedly reduced to 52%, 42%, and 28% of their predicted values, respectively.

**Figure 2:**
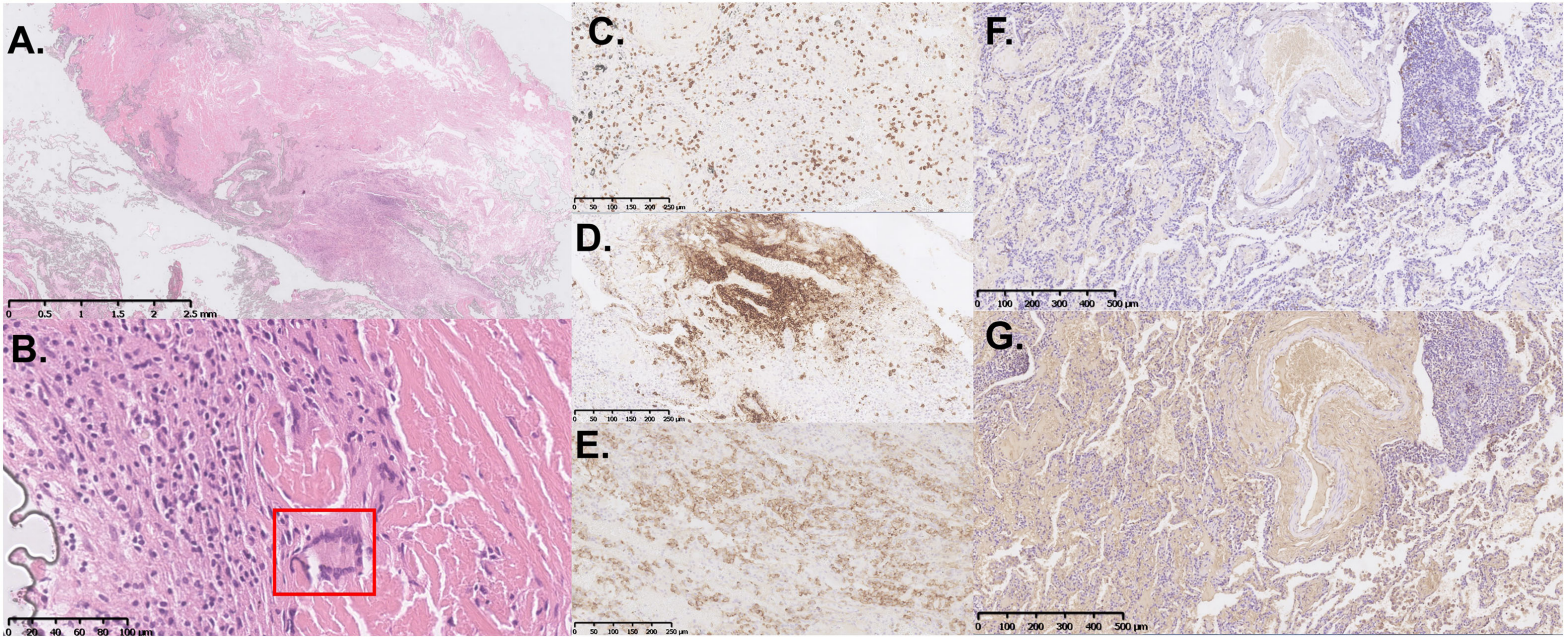
Histopathological and immunohistochemical features of the VATS lung biopsy specimen. **(A, B)** Representative hematoxylin and eosin (H&E)-stained sections show extensive destruction of lung tissue architecture, with large, homogeneous, eosinophilic material deposition. Inflammatory infiltrates composed of lymphocytes and plasma cells are present, accompanied by multinucleated giant cell reactions [indicated by the red box in **(B)**]. Immunohistochemical staining is positive for CD3 **(C)** and CD20 **(D)**. **(E)** CD138 immunohistochemical staining highlights numerous infiltrating plasma cells. Immunohistochemical staining for light chains showed λ negativity **(F)** and κ positivity **(G)**.

The histological sections obtained two years ago were reviewed, revealing destruction of lung tissue architecture (**Figures 2A, B**). Large areas of homogeneous cloud-like eosinophilic material lacking characteristic birefringence under Congo red staining observed by polarized light microscopy were noted. Significant infiltration of lymphocytes and plasma cells, accompanied by multinucleated giant cell reactions (indicated by the red box in **Figure 2B**), was found in the surrounding areas. The involved vascular walls exhibited deposition of eosinophilic material and infiltration of inflammatory cells, including lymphocytes (CD3- and CD20-positive) (**Figures 2C, D**) and plasma cells (CD138-positive) (**Figure 2E**), which resulted in partial luminal occlusion. Furthermore, immunohistochemical staining for light chains showed λ negativity (**Figure 2F**) and κ positivity (**Figure 2G**). These findings are consistent with the distinctive pathological features associated with light chain deposition disease (5).

**Figure 3:**
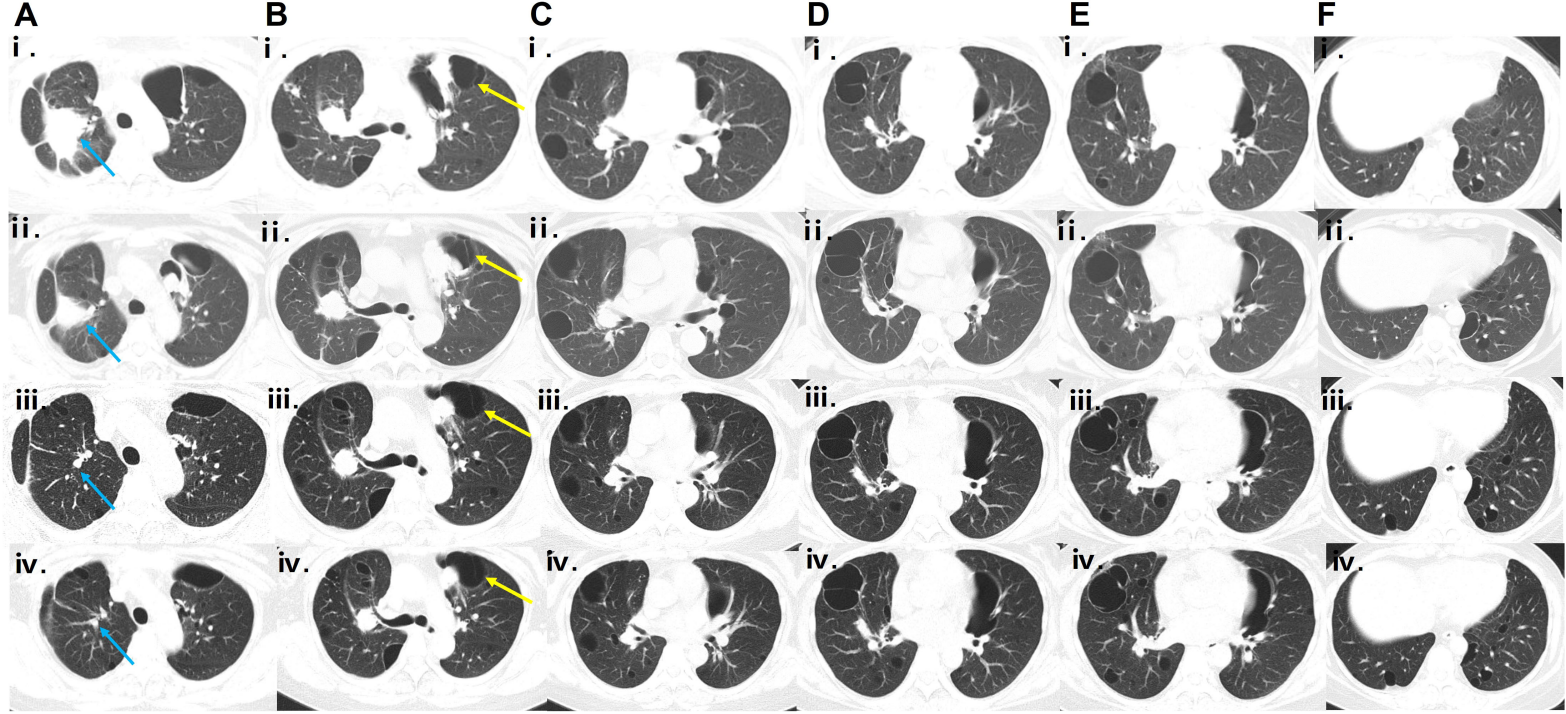
Serial CT scans during follow-up. **(Ai-Fi, Aii-Fii)** Chest CT scans obtained in June 2021 and February 2022, respectively, showing a shrinking trend of the nodule in the right upper lobe (the location indicated by the blue arrow), with dimensions decreasing from 51.8 mm × 42.3 mm to 30.0 mm × 25.5 mm. **(Aiii–Fⅲ, Aiv–Fiv)** HRCT scans obtained in July 2023 and September 2024, respectively, showing shrinkage of multiple cysts, especially the left subpleural cyst (indicated by the yellow arrow; serial dimensions from June 2021 to September 2024: 74.8 mm × 54.6 mm → 40.5 mm × 36.5 mm → 58.0 mm × 33.1 mm → 56.9 mm × 31.7 mm), and continued reduction of nodules, particularly the large nodule in the right upper lobe (indicated by the blue arrow; serial dimensions from June 2021 to September 2024: 51.8 mm × 42.3 mm → 30 mm × 25.5 mm → 7.2 mm × 6.8 mm → 5.9 mm × 3.7 mm) after the bortezomib-based regimen compared with previous scans.

**Figure 4:**
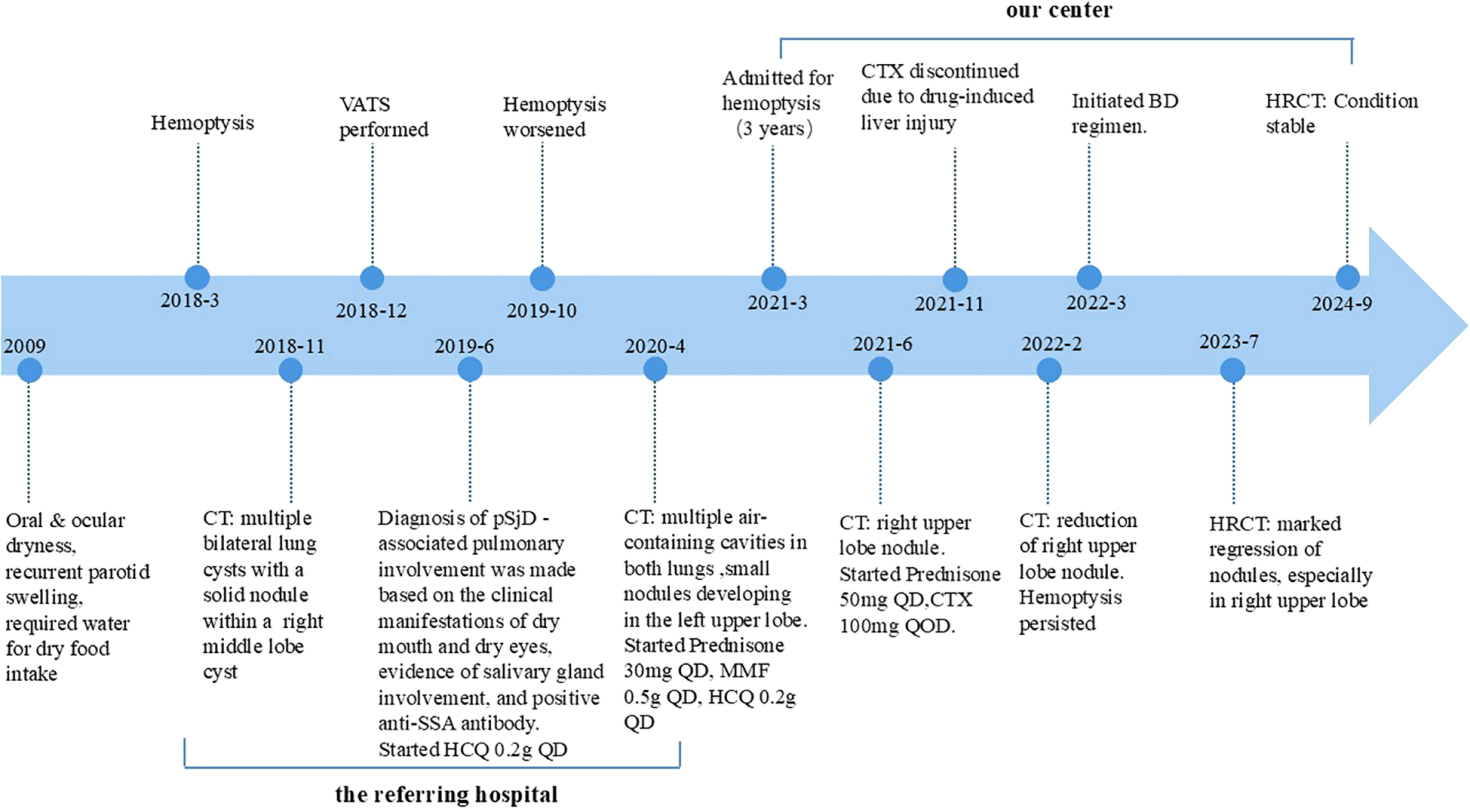
Timeline summarizing the patient’s diagnostic and treatment progression.

Based on her clinical presentation, radiological features, and pathological findings, the diagnosis of PLCDD secondary to pSjD was established. In June 2021, a routine follow-up chest CT scan revealed a nodule in the right upper lobe (**Figure 3Ai**). Then the patient received a therapeutic regimen for pSjD consisting of prednisone at a dose of 50 mg daily and cyclophosphamide (CTX) at a dose of 100 mg every other day. However, CTX had to be discontinued due to elevated ALT levels. Despite repeated chest CT scans revealed multiple large bullae persisted in both lungs, and the nodule in the right upper lobe demonstrated a shrinking trend (**Figures 3Aii-Fii**), the patient reported persistent daily hemoptysis. Following a multidisciplinary team (MDT) discussion, the administration of bortezomib in combination with dexamethasone (BD regimen) was initiated in March 2022. Bortezomib was administered at a dose of 2.4 mg (1.3 mg/m^2^), and dexamethasone at a dose of 40 mg, weekly for a duration of 36 weeks (9 months), followed by a tapering to once every two weeks for 64 weeks (15 months). After weeks of treatment, the hemoptysis resolved. After 16 months following treatment initiation, repeat CT scans performed in July 2023 revealed notable shrinkage in multiple cysts and nodules, particularly the large nodule in the upper lobe of the right lung (**Figures 3Aiii-Fiii**). A follow-up CT scan in September 2024 (2.5 years after the regimen) indicated stable disease (**Figures 3Aiv-Fiv**). The patient remained clinically stable with only occasional blood-streaked sputum. She is currently undergoing long-term follow-up (**Figure 4**).

## 3 Discussion

LCDD belongs to the category of immunoglobulin deposition disorders. It is characterized by accumulation of non-amyloid immunoglobulin light chains, which can result in functional impairment in various organs. Kidneys are predominantly affected, followed by liver and heart, while pulmonary involvement is extremely rare, with fewer than one hundred cases reported since its initial description in 1988 (6).

The etiology of PLCDD is not well established. Since the primary pathogenic factors of PLCDD are the light chain segments, the activation and proliferation of B-lymphocytes that produce immunoglobulin light chains may be critical in development of PLCDD. Therefore, PLCDD often occurs as a secondary condition to lymphoproliferative disorders or certain autoimmune diseases such as pSjD. Additionally, impaired degradation of immunoglobulin light chains and changes in the microenvironment of the lung tissue may contribute together to the accumulation of light chain materials.

The clinical manifestations of PLCDD are diverse. Patients often remain asymptomatic when the immunoglobulin light chains deposit as benign pulmonary nodules. Additionally, patients with nodular PLCDD may present with several nonspecific symptoms such as cough, dyspnea, and chest pain. Patients may also present with systemic hyperinflammatory states, such as fatigue, low-grade fever, night sweats, and weight loss.

Under certain circumstances, the recruitment and activation of macrophagic giant cells by immunoglobulin light chains can induce cystic lung destruction. These cells are known to degrade the extracellular matrix through matrix metalloproteinases (MMPs). Patients with cystic PLCDD experience more severe dyspnea and may progressively develop respiratory failure. Importantly, MMPs can also disrupt blood vessel integrity, potentially leading to hemoptysis when pulmonary blood vessels are affected (2).

The clinical presentations of PLCDD are atypical, so radiology and pathology play important roles in diagnosing PLCDD. Typical CT features of PLCDD include diffuse cysts with vascular shadows crossing through the cysts and multiple irregular pulmonary nodules with variable sizes and densities, occasionally with calcification. If pulmonary blood vessels are involved, contrast-enhanced CT may reveal vascular tortuosity and thickening, along with calcifications around small blood vessels. In patients with pSjD, lymphocytic interstitial pneumonia (LIP) and follicular bronchiolitis (FB) are the most common differential diagnoses for cystic lung lesions. Although both may present with thin-walled cysts, they are typically characterized by diffuse centrilobular nodules or ground-glass opacities. Histopathologically, the distinction is even more pronounced: LIP and FB are defined by dense, polyclonal lymphocytic infiltration and well-developed lymphoid follicles. In contrast, the present case exhibited extensive monoclonal light chain deposition (κ restriction) without lymphoid follicle formation, which is the key pathognomonic feature distinguishing PLCDD from LIP and FB. In women of childbearing age presenting with cystic lung parenchymal destruction, differential diagnosis from lymphangioleiomyomatosis (LAM) is required. LAM typically manifests on high-resolution CT (HRCT) as numerous, thin-walled, and well-defined round cysts that are diffusely and uniformly distributed throughout both lungs, often accompanied by complications such as spontaneous pneumothorax or chylothorax. However, the presence of recurrent hemoptysis and multiple pulmonary nodules in this patient is inconsistent with the typical phenotype of LAM and cannot be explained by its established pathophysiology. In middle-aged female patients presenting with multiple pulmonary nodules, sarcoidosis is a critical differential diagnosis. However, sarcoidosis typically presents with perilymphatic micronodules with irregular borders and a predominantly symmetric distribution in the upper and middle lung fields, frequently accompanied by mediastinal and bilateral hilar lymphadenopathy. Clinically, hemoptysis is uncommon, and histopathological examination characteristically reveals non-caseating granulomas, which usually shows a favorable response to corticosteroid therapy. These features are inconsistent with the clinical and radiological findings in our patient. With regard to fungal infection, particularly aspergillosis, the presence of intracavitary nodular opacities warrants consideration. Aspergillosis commonly manifests as ovoid, intracavitary nodules with heterogeneous density that exhibit gravity-dependent movement within the cavity. The surrounding cavities typically have thick, irregular walls. Furthermore, active fungal infections often progress radiologically during prolonged corticosteroid use. In contrast, our patient remained stable without deterioration under systemic steroid therapy, showed no radiological progression and had negative histopathological results, including absence of fungal elements on special stains such as Gomori methenamine silver (GMS) and periodic acid–Schiff (PAS)—further arguing against an infectious etiology.

Considering the difficulty of radiological examination in differentiating PLCDD from other pulmonary cystic diseases, such as pulmonary light chain amyloidosis (AL) and pulmonary Langerhans’ cell histiocytosis, pathological findings are critical for confirming the diagnosis of PLCDD. Therefore, surgical biopsy is recommended for patients suspected of having PLCDD based on radiological findings. Histologically, PLCDD often presents as lung tissue destruction with the formation of single or multiple nodules and cysts. These nodules consist of abundant amorphous eosinophilic material that resembles amyloid but is Congo red-negative under polarized light microscopy. The eosinophilic material often involves vessel walls and occasionally affects bronchial walls. Infiltration of polyclonal lymphocytes, plasma cells, and multinucleated giant cells can be observed surrounding the nodules and cysts. Upon detecting pulmonary light chain deposition, it is imperative to differentiate whether the pathology is secondary to an autoimmune disorder, such as Sjögren disease in this case or driven by an underlying plasma cell dyscrasia. In this patient, the systemic screening for CRAB criteria (Hypercalcemia, Renal insufficiency, Anemia, and Bone lesions) yielded negative results, thereby effectively ruling out a diagnosis of Multiple Myeloma (MM).

Due to the limited number of cases, the management strategy for PLCDD is not definitely established. However, the general principle has been determined as blocking the production of immunoglobulin light chains. Considering that primary diseases play a crucial role in activating and proliferating light chain-producing B cells, glucocorticoid accompanied by immunosuppressive agents have commonly been used to target these primary diseases. Additionally, insights from other disorders that belong to the same disease category can also be applied in treating PLCDD. AL is an immunoglobulin deposition disorder that shares the same category with PLCDD. The therapeutic guidelines for AL recommend bortezomib-based regimens as first-line treatment (7) Furthermore, this therapy has also shown reasonable efficacy in renal-involved LCDD (8, 9). However, their efficacy in PLCDD remains uncertain, with only one case report describing the use of bortezomib in a PLCDD patient preceding autologous stem cell transplantation (10).

Bortezomib is a proteasome inhibitor first approved for multiple myeloma (11). Its major mechanism is to target the ubiquitin-proteasome system that induce endoplasmic reticulum stress related apoptosis on plasma cells. By depleting both short-lived and long-lived plasma cells, bortezomib has been shown efficacy in various antibody-mediated disease, including refractory pSjD, refractory SLE, thrombotic thrombocytopenic purpura, and among others (12). As monoclonal plasma cells serve as the source of immunoglobulin light chains in LCDD for patients with a background of autoimmune diseases, bortezomib might target this group of cells for the therapeutic intervention of it.

The patients in our case showed a good response in both clinical and radiological manifestation after BD treatment Therefore, BD regimen might potentially be effective in managing PLCDD. However, a higher level of evidence is required to confirm the efficacy of BD therapy in the treatment of PLCDD. In conclusion, PLCDD is a rare disorder whose therapeutic strategy has not been established yet. This case presentation indicated that targeted therapy with bortezomib may provide a promising approach for refractory disease, particularly those associated with underlying autoimmune diseases.

## 4 Patient perspective

Living with this illness for years, the recurrent hemoptysis filled me with immense fear and uncertainty. After several treatments failed to bring lasting improvement, I had nearly lost hope. Starting the combination therapy of bortezomib and dexamethasone marked a turning point in my battle. To my immense relief, the hemoptysis that had plagued me for so long resolved within weeks, which was the first sign of real progress. As the treatment continued, my breathing improved significantly, allowing me to gradually return to my daily activities. I am deeply grateful to my medical team for their dedication and for pursuing this targeted treatment. While the long course of therapy was challenging, witnessing the shrinkage of the cysts and nodules on my follow-up CT scans made every step worthwhile. Now, with my condition stable, I feel a renewed sense of hope for the future. I sincerely hope that sharing my experience can offer encouragement to other patients facing similar rare diseases.

## Data Availability

The original contributions presented in the study are included in the article/supplementary material. Further inquiries can be directed to the corresponding author.
